# Comparative Efficacy of Fixed Versus Removable Habit-Breaking Appliances for the Management of Non-nutritive Sucking Habits: A Systematic Review

**DOI:** 10.7759/cureus.99043

**Published:** 2025-12-12

**Authors:** Bandar M Barnawi, Wejdan A Al Rashidi, Rawan A Al Qahtani, Najem A Alotaibi, Abdulqader Hussain A Hejji, Afnan H Balkhy, Bashaer Aidhah S Al Ghashmari, Bashair M Baqies, Rashed A Alghamdi, Nawaf A Alsaedi, Danah S Basaad, Maha A Alzahrani

**Affiliations:** 1 Family Dentistry, Al Hizam Primary Health Care, Madinah Health Cluster, Ministry of Health, Medina, SAU; 2 Dentistry, Majmaah University, Majmaah, SAU; 3 Dentistry, King Saud Bin Abdulaziz University for Health Sciences, Riyadh, SAU; 4 General Dentistry, King Abdullah Bin Abdulaziz University Hospital, Jeddah, SAU; 5 Dentistry, Jazan University, Jazan, SAU; 6 General Dentistry, Al Takhasosi Primary Health Care Center, Makkah Health Cluster, Ministry of Health, Makkah, SAU; 7 General Dentistry, King Abdulaziz University, Jeddah, SAU; 8 General Dentistry, King Fahad Hospital, Al Baha Health Cluster, Ministry of Health, Al Baha, SAU; 9 Dentistry, Al Baha University, Al Baha, SAU; 10 Pediatric Dentistry, East Riyadh Dental Centre, Second Riyadh Cluster, Ministry of Health, Riyadh, SAU; 11 Restorative Dentistry, East Riyadh Dental Centre, Second Riyadh Cluster, Ministry of Health, Riyadh, SAU

**Keywords:** fixed appliance, habit-breaking appliance, non-nutritive sucking, palatal crib, removable appliance

## Abstract

Non-nutritive sucking habits, including thumb, finger, and pacifier sucking, are common self-soothing behaviors in early childhood, and persistence beyond the preschool years is associated with anterior open bite, increased overjet, and altered orofacial function. Interceptive management often involves habit-breaking appliances that may be fixed or removable, yet their relative effectiveness remains debated. This systematic review compared the clinical efficacy of fixed versus removable habit-breaking appliances in achieving habit cessation and correcting associated dentoalveolar discrepancies in children. Electronic searches covering January 2005 to October 2025 identified randomized and prospective clinical studies evaluating these appliances. Outcomes of interest included overbite and overjet changes, skeletal and dentoalveolar effects, habit-cessation rates, and adverse events. A total of 15 studies met the inclusion criteria. Fixed appliances, particularly palatal cribs, bonded spurs, and Bluegrass designs, consistently produced faster overbite correction, with mean gains of about 3.0-3.6 mm, and better vertical control than removable devices, while removable appliances achieved comparable improvements mainly when compliance was high. Functional assessments also showed improved tongue posture and pressure patterns following treatment. Reported adverse effects were generally mild and transient, most commonly temporary speech alteration and local irritation. Overall, fixed appliances provide more predictable and efficient correction of malocclusions related to non-nutritive sucking habits, whereas removable appliances can be similarly effective when patient cooperation is strong, supporting early and individualized appliance selection to optimize stability and reduce relapse.

## Introduction and background

Non-nutritive sucking habits (NNSHs), which include thumb, finger, or pacifier (dummy) sucking, are common self-soothing behaviors in young children [1]. Prevalence studies suggest that a substantial proportion of infants and toddlers exhibit some form of digit or pacifier sucking, regardless of socioeconomic status. These habits engage the infant’s natural sucking reflex and are considered a normal part of development in the early years. In most cases, children outgrow these habits naturally, typically by two to four years of age [2]. However, when sucking habits persist beyond the normal developmental period, they can have negative effects on the child’s developing dentition and facial growth. Longitudinal cohort studies have consistently demonstrated that children who continue NNSHs into the mixed dentition phase are at greater risk of malocclusions such as anterior open bite, increased overjet, and posterior crossbite [3]. The prevalence of these dental anomalies correlates directly with both the duration and intensity of the sucking habit [4]. From a biomechanical standpoint, the repetitive forces exerted by a digit or pacifier on the anterior maxillary segment and palatal vault induce proclination of maxillary incisors and retroclination of mandibular incisors, thereby increasing overjet and anterior open bite tendency [5]. The biological plausibility of this mechanism has been confirmed in experimental and cephalometric studies showing that even low-magnitude forces applied consistently over time are sufficient to remodel alveolar bone and alter tooth position [6]. Apart from dentoalveolar effects, prolonged sucking may also interrupt the functional equilibrium of the orofacial musculature, resulting in altered tongue posture, atypical swallowing, and compromised speech articulation. The combination of skeletal, dental, and functional disturbances underscores the multifactorial nature of these developmental alterations and the necessity for early orthodontic interception.

Etiologically, NNSH persistence is influenced by behavioral, emotional, and environmental factors. Studies in child psychology have suggested that continued thumb or pacifier use may serve as an anxiety-relieving behavior, which is reinforced during periods of stress or parental absence. Epidemiological data further indicate that feeding practices and caregiver responses influence both onset and persistence of NNSH. These findings are supported by population-based research showing that breastfeeding beyond six months significantly reduces the risk of developing malocclusions associated with NNSHs [7]. Conversely, prolonged use of pacifiers, beyond age three, has been independently linked to increased incidence of open bite and crossbite in both primary and mixed dentition [8]. The psychosocial context of these habits further complicates management. Parental tolerance, socioeconomic status, and cultural attitudes can either reinforce or discourage the persistence of the behaviors [9]. Warren et al. observed that NNSHs often persist longer when parents view the habits as harmless, suggesting that early parental education may prevent the development of malocclusion [10]. Due to its multifactorial nature, NNSH necessitates a multidisciplinary approach to management, encompassing behavioral modification, parental counseling, and mechanical intervention.

Mechanical interruption of the habit is indicated when behavioral techniques fail or when malocclusion has already developed. Habit-breaking appliances (HBAs) constitute the principal orthodontic modality for intercepting persistent NNSH and correcting associated malocclusions. These appliances are broadly classified as fixed or removable according to their retention and mode of action. Fixed appliances include the palatal crib, Bluegrass appliance, and bonded spurs. These are cemented intraorally and remain active continuously, preventing the finger or pacifier from achieving suction against the palate. They act primarily as physical barriers, removing both the tactile and psychological gratification derived from sucking. In contrast, removable appliances function intermittently and depend heavily on patient cooperation. While both appliance designs share the same therapeutic aim, their practical effectiveness is influenced by distinct behavioral and mechanical factors. Fixed appliances eliminate the variable of compliance and tend to produce faster and more consistent results, especially in younger or uncooperative patients. Removable devices, however, offer advantages such as hygiene maintenance, comfort, and aesthetics. They are often preferred for motivated older children. Despite these clinical distinctions, evidence comparing the relative efficacy of the two appliance categories remains inconclusive. Some studies report higher cessation rates and improved occlusal outcomes with fixed appliances, whereas others find that removable appliances can achieve comparable results when compliance is adequate [11].

From a functional standpoint, the cessation of NNSH, regardless of appliance type, permits re-establishment of orofacial muscle balance and facilitates spontaneous correction of anterior open bite through incisor extrusion and normalization of tongue posture [12]. However, incomplete cessation or premature appliance removal frequently results in relapse. Even after mechanical correction, stability can be undermined by persistent dysfunctional habits such as abnormal tongue posture and swallowing. Consequently, adjunctive myofunctional therapy is often considered to reinforce normal patterns following appliance removal, although more robust evidence is needed to confirm its efficacy [13]. Despite widespread clinical use, studies on HBAs have significant methodological variability, limiting the ability to draw definitive conclusions regarding the superiority of one modality over another. Differences in patient age, diagnostic criteria for open bite, treatment duration, and outcome assessment are some of the factors that contribute to heterogeneous findings. Furthermore, many studies measure only morphological parameters such as overbite and overjet without quantifying actual habit cessation or relapse rates [14]. In addition, there has been a broader shift toward individualized, behaviorally informed care in pediatric dentistry. For example, Almarshadi et al. (2025) report that habit-breaking orthodontic devices can be 3D-printed to precisely match a patient’s anatomy, significantly improving their fit, comfort, and effectiveness [15]. Meanwhile, electronic reminder devices that deliver gentle vibratory feedback when the digit contacts the lips or mouth have been piloted as adjuncts to traditional methods. Given these developments, a critical synthesis of comparative evidence is needed to clarify the relative advantages of fixed and removable HBAs in eliminating NNSH and correcting associated malocclusions.

Accordingly, the present review was designed with a primary focus on short-term change in overbite as the main clinical outcome, and secondary outcomes including habit cessation, long-term post-treatment stability, and any reported adverse effects of treatment. Despite the abundance of clinical reports, the heterogeneity of existing studies and the absence of standardized evaluation frameworks hinder meaningful comparisons. Therefore, the present systematic review aims to comprehensively evaluate and compare the efficacy of fixed versus removable HBAs for the management of NNSHs in children, focusing on habit cessation, occlusal correction, and treatment stability.

## Review

Methodology

Information Sources and Search Strategy

The PECO framework guided the formulation of the research question for this systematic review [16]. An electronic search was undertaken in Google Scholar, Scopus, and PubMed/MEDLINE, using combinations of keywords related to HBAs and non-nutritive sucking behaviors. The finalized search terms were as follows: “non-nutritive sucking” OR “thumb sucking” OR “finger sucking” OR “digit sucking” OR “pacifier use” OR “oral habit,” combined with “habit-breaking appliance” OR “habit breaker” OR “palatal crib” OR “palatal arch” OR “bonded spur” OR “Bluegrass appliance” OR “orthodontic appliance,” and further limited by “fixed appliance” OR “removable appliance” OR “comparative” OR “randomized controlled trial.” The search was restricted to English-language human studies published from January 2005 to October 2025. This twenty-year time span was chosen to capture contemporary orthodontic techniques and exclude outdated appliance designs.

Study Selection

All retrieved titles and abstracts were screened independently by two reviewers to determine eligibility according to predefined inclusion and exclusion criteria. Full-text articles of potentially relevant studies were obtained and examined in detail to assess for inclusion. Discrepancies in study selection were resolved by discussion and, where necessary, by consultation with a third reviewer in accordance with the Preferred Reporting Items for Systematic Reviews and Meta-Analyses (PRISMA) 2020 guidelines [17].

Data Collection and Data Items

A standardized, pre-piloted data extraction form was developed and tested on four representative studies to ensure consistency. The following information was extracted from each eligible article: first author, year and country of publication, study design, sample characteristics, type and duration of interventions, outcome measures (habit cessation, relapse, overbite or overjet changes, and adverse effects), and principal findings.

Eligibility Criteria

Studies were included if they investigated children with NNSHs (thumb, finger, or pacifier use) managed with intraoral HBAs (fixed, removable, or both) and reported at least one measurable clinical or behavioral outcome. For the primary comparison of interest, we focused on studies that directly compared at least one fixed appliance with at least one removable habit-breaking device. In addition, studies evaluating only fixed appliances, only removable appliances, or comparisons with untreated or normal-occlusion controls were also included when they provided relevant data on habit cessation, overbite correction, treatment stability, or related functional outcomes. Eligible study designs comprised randomized controlled trials (RCTs), controlled clinical trials, and comparative cohort studies. Studies were excluded if they involved no intraoral HBA; were case reports, reviews, or non-clinical investigations; or were published in languages other than English. Articles focusing solely on unrelated oral habits, such as tongue thrusting, or on appliance design without patient data, were also excluded.

Risk of Bias Assessment

Two reviewers independently assessed the risk of bias for each study. For RCTs, the Cochrane Risk of Bias 2 (RoB 2) tool [18] was employed, evaluating domains such as the randomization process, deviations from intended interventions, missing outcome data, outcome measurement, and selection of reported results. Each RCT was judged as “low risk,” “some concerns,” or “high risk” of bias. For non-randomized studies, the Risk of Bias in Non-randomized Studies of Interventions (ROBINS-I) tool [19] was implemented, assessing bias due to confounding, participant selection, classification of interventions, deviations from intended interventions, missing data, outcome measurement, and selection of reported results. These were rated as “low,” “moderate,” “serious,” or “critical” risk of bias. Discrepancies were resolved by a third reviewer.

Certainty of Evidence (GRADE)

Certainty of evidence was assessed for each outcome using the GRADE approach [20]. Evidence from randomized trials started at high certainty and observational studies at low certainty, and this was downgraded as appropriate for risk of bias, inconsistency, indirectness, imprecision, and publication bias. Two reviewers independently performed GRADE ratings and reached a consensus through discussion; disagreements, when present, were resolved by a third reviewer. The overall certainty was categorized as high, moderate, low, or very low, and it is presented alongside outcomes in the Results section.

Effect Measures and Synthesis Methods

A meta-analysis was performed using a random-effects model to compare fixed versus removable HBAs [21]. Only studies that directly compared a fixed appliance group to a removable appliance group in children with NNSH-related anterior open bite were included in the quantitative synthesis. Studies involving adjunctive therapies or lacking comparable outcome measures were excluded from the meta-analysis. The primary outcome pooled was the change in overbite after treatment. Habit cessation rates and other outcomes were collected when available for descriptive analysis, but they were not meta-analyzed due to inconsistent reporting. The weighted mean differences (fixed minus removable) with 95% confidence intervals (CIs) were calculated for overbite closure, and the I² statistic was used to assess statistical heterogeneity [22]. All meta-analyses were conducted in accordance with PRISMA guidelines [17] using RevMan 5.4 [23].

Reporting Bias Assessment

Due to the limited number of studies included in the quantitative analysis (n < 10), a formal assessment of publication bias (e.g., funnel plot or Egger’s test) was not performed.

Results

**Figure 1:**
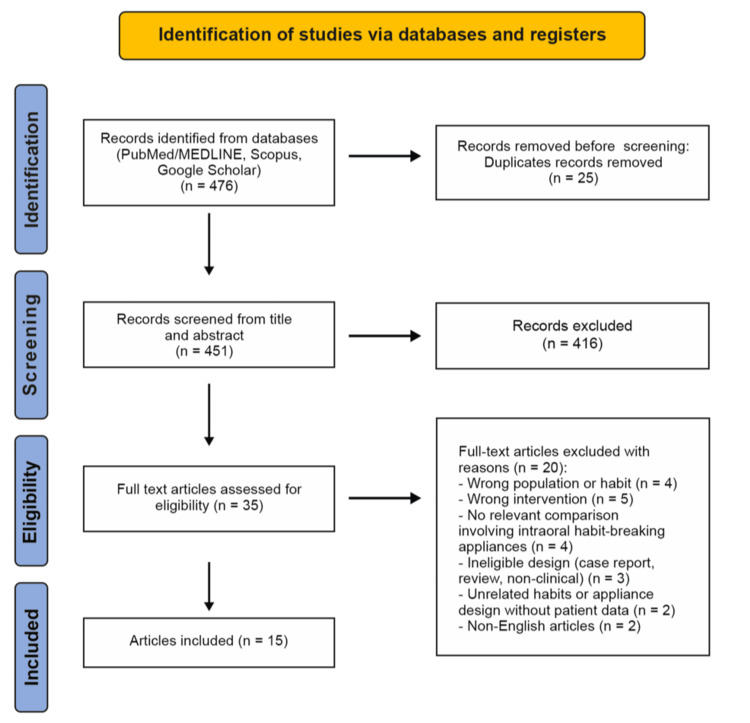
Preferred Reporting Items for Systematic Reviews and Meta-Analyses (PRISMA) 2020 flow diagram.

Following PRISMA 2020 guidance, the database search yielded 476 records from PubMed/MEDLINE, Scopus, and Google Scholar. After removal of 25 duplicates, 451 titles and abstracts were screened, of which 416 were excluded as they were not relevant. The remaining 35 full-text articles were assessed for eligibility, and 20 were excluded for predefined reasons. Ultimately, 15 studies met the inclusion criteria and were incorporated into the qualitative synthesis. These trials were published between 2005 and 2025 and represented a range of geographical settings and study designs, including RCTs, controlled clinical studies, and prospective cohort investigations. Figure 1 summarizes the flow of records through the identification, screening, eligibility, and inclusion stages.

The characteristics of the 15 included studies are summarized in Table 1, including country and setting, study design, sample size and age, habit type, appliance protocols and comparisons, follow-up duration, and key outcomes and methodological notes.

**Table 1:** Characteristics of included studies. OB: overbite; OJ: overjet; AOB: anterior open bite; PP–MPA: palatal plane–mandibular plane angle; QH: quad helix; C: chin cup; HP: high-pull; HG: headgear; S: spurs; SBU: spurs with bite build-ups; RPBP/C: removable posterior bite plane + crib; TPA: transpalatal arch; FS: functional score; OMT: orofacial myofunctional therapy; Tx: treatment; NS: not significant; USG: ultrasonography; LB: lip bumper

Study	Country/Setting	Design	Sample (n; age)	Habit/Condition	Appliance(s) and comparison (fixed vs. removable)	Follow-up (months)	Outcomes collected	Notes
Canuto et al. (2016) [24]	Brazil	Comparative cohort (3-arm)	68 total; mean age ~9.3 years. Groups: 20 bonded lingual spurs (BLS), 21 conventional lingual spurs (CLS), 27 untreated controls	AOB (mixed dentition)	Bonded vs. conventional lingual spurs (fixed vs. conventional) + control	~12	Craniofacial and dentoalveolar (OB, ceph)	Isolated effects of spur type
Cassis et al. (2012) [25]	Brazil	Prospective cohort (treated vs. control)	30 treated; 30 controls; mean age ~8 years	Class I AOB	Bonded spurs + high-pull chincup (fixed adjunct) vs. untreated controls	12	Ceph angles, OB	Dentoalveolar-driven OB increase
Cassis et al. (2018) [26]	Brazil	Longitudinal stability cohort	25 treated; 23 normal-occlusion controls	Class I AOB	Bonded spurs + high-pull chincup (stability follow-up)	36 post-tx	Overbite stability	96% clinical stability at 36 months
Cozza et al. (2006) [27]	Italy (university)	Controlled cohort	23 treated; 23 controls; ~8–10 years	AOB with thumb sucking	Quad-helix + crib (fixed) vs. untreated controls	~18 active	OB, ceph angles, lip profile	90% achieved positive OB; vertical improvement
Cozza et al. (2007) [28]	Italy	Controlled cohort (post-tx)	21 QH/Crib; 21 controls	Thumb sucking + dentoskeletal OB	QH/Crib (fixed) vs. controls	~24 post-tx	Skeletal/dental changes	Post-treatment effects retained
Eltager et al. (2025) [29]	Egypt	Pilot RCT	22 (6–14 years)	Thumb/finger NNSH	Electronic reminder (extraoral) vs. palatal crib (fixed)	6–9	Habit cessation; acceptability	Higher cessation in crib; NS difference
Giuntini et al. (2008) [30]	Italy	Controlled cohort	20 QH/Crib vs 20 removable plate + crib	Dentoskeletal AOB	QH/Crib (fixed) vs. removable crib plate	~18	Cephs, OB	Both improved AOB; pattern differences
Insabralde et al. (2016) [31]	Brazil	4-arm cohort + control	77 treated; 30 controls	AOB	Removable crib + CC; bonded spurs + CC; CC only; control	12	Cephs, OB	Barrier + CC best OB gain
Leite et al. (2016) [32]	Brazil	RCT (3 arms)	13 controls; 13 fixed crib; 13 bonded spurs	Early AOB	Fixed palatal crib vs. bonded spurs vs. control	6 & 12	OB, cephs	Both active arms effective
Mucedero et al. (2013) [33]	Italy	Long-term controlled cohort	28 QH/Crib; 20 controls	Dentoskeletal OB	QH/Crib (fixed) vs. controls	≥60 post-tx	Stability, vertical measures	Long-term stability shown
Rossato et al. (2018) [34]	Brazil	RCT (4 arms)	Final sample 81; mean age ~8.4 years	Mixed-dentition AOB with NNSH/tongue thrust	Bonded spurs; chincup; fixed crib; removable crib (comparative)	12	OB, dentoalveolar/ceph	Multi-arm head-to-head comparison
Sanguida et al. (2017) [35]	India	Pilot cohort	10 enrolled → 6 completers; 8–13 years	Digit sucking	Fixed anti-digit reminder appliance	6	USG muscle changes + OB	Small n; functional changes NS
Slaviero et al. (2017) [36]	Brazil	RCT	41 (7–10 years)	AOB	Fixed crib vs. removable crib	12	Arch dimensions (digital models)	Dimensional patterns differed
Torres et al. (2006) [37]	Brazil	RCT	30 treated; 30 controls	Class I AOB	Removable crib + high-pull CC vs no-tx	12	Cephs; soft tissues	AOB closure via dentoalveolar change
Torres et al. (2012) [38]	Brazil	Prospective cohort	30 Rpc + CC vs. 30 Fpc + CC	AOB (often NNSH-related)	Removable crib + CC vs. fixed crib + CC	12	OB, incisor inclination, molar changes	Fixed crib faster OB closure; removable better inclination control

Overall, both fixed and removable appliances were associated with improvements in anterior open bite. However, fixed appliances tended to produce changes more rapidly. Table 2 summarizes the outcomes of each study, including overbite changes and any adverse effects or patient-reported outcomes.

**Table 2:** Summary of outcomes for included studies. OB: overbite; OJ: overjet; AOB: anterior open bite; PP–MPA: palatal plane–mandibular plane angle; QH: quad helix; C: chincup; HP: high-pull; HG: headgear; S: spurs; SBU: spurs with bite build-ups; RPBP/C: removable posterior bite plane + crib; TPA: transpalatal arch; FS: functional score; OMT: orofacial myofunctional therapy; Tx: treatment; NS: not significant; USG: ultrasonography; LB: lip bumper

Study (first author, year)	Primary clinical endpoint(s)	Key quantitative outcome(s)	Patient-reported/Adverse events	Bottom-line signal (fixed vs. removable vs. other)
Canuto et al. (2016) [24]	Dentoalveolar overbite correction; spur acceptability	Both bonded and conventional lingual spurs produced similar, significant OB increases vs. control	Bonded spurs had better chewing/eating acceptance; most children adapted within ~1 week	Bonded vs. conventional spurs: both effective; bonded better tolerated
Cassis et al. (2012) [25]	Cephalometric change; overbite	OB ↑; palatal tipping of maxillary incisors; vertical incisor development	—	Spurs + high-pull chincup effective (dentoalveolar-driven change)
Cassis et al. (2018) [26]	Post-treatment stability (36 months)	96% clinical stability; minimal relapse	—	Good medium-term stability after fixed spurs + chincup
Cozza et al. (2006) [27]	Overbite correction; vertical skeletal relationships	+3.6 mm OB vs control; ↓ PP–MPA ~1.7°; ~90% positive OB	Not detailed	Fixed QH/Crib effective with vertical skeletal improvement
Cozza et al. (2007) [28]	Post-treatment effects	Retained anterior dental corrections	—	Post-treatment effects persist
Eltager et al. (2025, pilot) [29]	Habit cessation	27.3% (watch reminder) vs. 54.5% (palatal crib); NS	Gingival inflammation + transient speech interference in crib group, resolved with hygiene/adaptation	Fixed intraoral crib trends better cessation than extraoral reminder
Giuntini et al. (2008) [30]	Dento-skeletal AOB change	Both fixed and removable improved; different response patterns	—	Both useful; fixed protocol yields more robust OB change
Insabralde et al. (2016) [31]	OB and cephalometric outcomes	Removable crib + chincup and spurs + chincup > chincup only	—	Barrier + chincup best; chincup alone least effective
Leite et al. (2016) [32]	OB and cephalometric outcomes	Fixed crib and bonded spurs → positive OB; crib slight edge	—	Both fixed options effective; crib marginally stronger
Mucedero et al. (2013) [33]	Long-term stability	Maintained OB; reduced divergence long term	—	QH/Crib stable in long term
Rossato et al. (2018) [34]	Overbite change across 4 arms	Crib and spurs > chincup alone for OB	—	“Barrier” appliances outperform chincup monotherapy
Sanguida et al. (2017) [35]	Muscle function after cessation	Functional/muscle changes not statistically significant; small n	None notable	USG feasible to monitor muscle function post-cessation
Slaviero et al. (2017) [36]	Arch dimensional changes	Fixed vs removable crib: different arch-dimension patterns	—	Choose based on arch goals & compliance
Torres et al. (2006) [37]	Dentoalveolar change	OB closure mainly via incisor extrusion/retrusion	—	Removable crib + HP chincup works
Torres et al. (2012) [38]	OB and incisor inclination	Fpc + C > Rpc + C for OB gain (~+1.3 mm via maxillary incisor extrusion); Rpc + C better incisor inclination control	—	Fixed crib + chincup closes OB faster; removable better for proclination control

Risk of Bias in Studies

Using the RoB 2 tool for RCTs, none of the randomized trials were deemed low risk of bias. Most were judged as having “some concerns,” mainly due to lack of blinding (the appliance type was evident to patients and assessors) and, in a few cases, unclear allocation concealment or incomplete outcome data. One small pilot trial had a high risk of bias because of methodological limitations. For the non-randomized studies, ROBINS-I assessments indicated moderate to serious risk of bias in nearly all cases, with common issues including confounding, selection bias, and measurement bias. These limitations reduce confidence in the individual study results and were considered in grading the overall evidence.

In addition to qualitative synthesis, quantitative data from two eligible studies reporting comparable overbite outcomes were pooled to assess the overall treatment effect. Mean differences, 95% CIs, and direction of effect are presented in Table 3. Studies included in the systematic review but not contributing to the quantitative synthesis were excluded from the meta-analysis if they lacked extractable data for overbite change, did not directly compare fixed and removable appliances, or assessed secondary or post-treatment outcomes such as long-term stability or muscle function.

**Table 3:** Quantitative summary of studies included in the meta-analysis comparing fixed and removable habit-breaking appliances. RCT: randomized controlled trial; CI: confidence interval; Tx: treatment group; Ctrl: control group

Study	Design	n (Tx)	n (Ctrl)	Appliance type	Mean difference in overbite (mm)	95 % CI (lower–upper)	Direction of effect
Giuntini et al. (2008) [30]	Controlled cohort	20	20	Fixed vs removable	-1.00	-2.06–0.06	Favors removable
Slaviero et al. (2017) [36]	RCT	21	20	Fixed vs removable crib	-0.37	-1.66–0.92	Favors removable
Pooled effect (95 % CI)	—	—	—	—	-0.75	-1.57–0.07	

**Figure 2:**
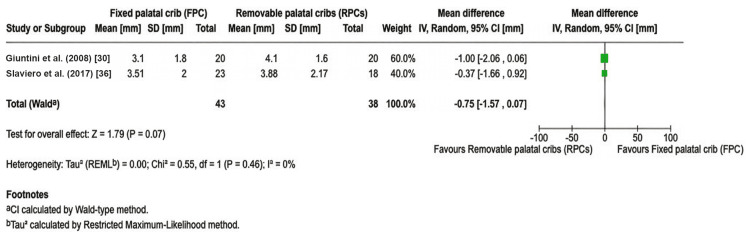
Forest plot comparing overbite improvement (mm) with fixed vs. removable habit-breaking appliances.

A meta-analysis of two studies (n = 81) comparing overbite correction achieved through fixed and removable HBAs revealed a pooled mean difference of −0.75 mm (95% CI = −1.57 to 0.07; p = 0.07), indicating that removable appliances produced marginally greater overbite gain than fixed appliances, though this difference did not achieve statistical significance. The Giuntini et al. (2008) cohort study demonstrated a mean difference of −1.0 mm (95% CI = −2.06 to 0.06), suggesting removable plate with crib (RP/C) produced greater overbite change than the quad-helix with crib (Q-H/C) appliances [30]. Conversely, the Slaviero et al. (2017) RCT showed a smaller mean difference of −0.37 mm (95% CI = −1.66 to 0.92), with removable appliances, demonstrating slightly superior numerical outcomes [36]. The statistical test for overall effect yielded a Z-statistic of 1.79 with a corresponding p-value of 0.07. A p-value of 0.07 (higher than the conventional significance threshold of 0.05) indicates that the result did not achieve statistical significance. However, this borderline p-value (just above the 0.05 cutoff) suggests that the finding approaches statistical significance and warrants careful interpretation. Heterogeneity assessment using the restricted maximum-likelihood method revealed a Tau² of 0.00, indicating zero between-study variance. The chi-square test for heterogeneity yielded a result of 0.55 with 1 degree of freedom and a p-value of 0.46, which was not statistically significant. The I² statistic, representing the percentage of total variation attributable to heterogeneity between studies, was 0%, indicating the absence of heterogeneity. Figure 2 presents the pooled comparison of overbite improvement between fixed and removable HBAs from the two available studies.

In Figure 2, each horizontal line represents one study’s mean overbite improvement difference (fixed minus removable) with its 95% CI. The solid squares denote the point estimates for each study (proportional to study weight), and the diamond represents the pooled mean difference. The vertical dashed line at 0 indicates no difference between groups. The pooled random-effects estimate (black diamond) demonstrates a mean difference of -0.75 mm (95% CI = -1.57 to 0.07) favoring removable appliances, although the 95% CI crossed the null line (zero), indicating statistical non-significance (p = 0.07). Both individual studies showed negative mean differences, with Giuntini exhibiting a stronger trend (MD -1.0 mm; 60.0% weight) compared to Slaviero (MD = -0.37 mm; 40.0% weight). The vertical dashed line at zero represents no difference between appliance types. Heterogeneity was negligible (I² = 0%, p = 0.46), indicating high concordance between studies. At the bottom of the forest plot, a large diamond represents the pooled result of the meta-analysis, positioned at -0.75 mm.

One noticeable observation in the forest plot is that the diamond is narrower than the individual study CIs because combining the two studies increases the overall statistical power and precision. However, despite this improvement in precision, the pooled CI still crosses the zero line, extending slightly into positive territory. This critical observation demonstrates that even when combined, the two studies do not provide statistically significant evidence that one appliance type produces greater overbite correction than the other. The p-value of 0.07 confirms the statistical non-significance of the combined estimate.

However, this quantitative finding merits contextualization within broader clinical evidence. Giuntini et al.’s investigation reported clinical success rates of 90% for fixed versus 60% for removable appliances (p < 0.001), suggesting that while removable appliances may achieve numerically larger mean overbite changes, fixed appliances demonstrate superior treatment consistency and predictability. Consequently, appliance selection should be individualized based on patient compliance capacity, age, skeletal characteristics, and esthetic considerations rather than anticipated overbite correction magnitude alone.

Certainty of Evidence

**Figure 3:**
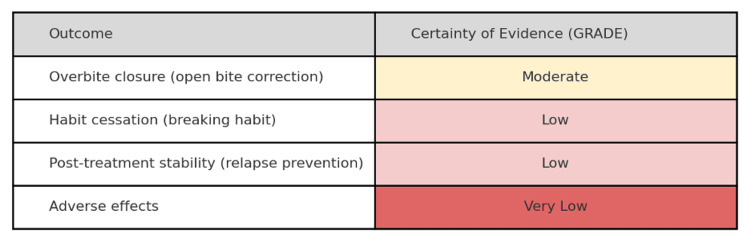
GRADE certainty of evidence for key outcomes. Each outcome is rated as high, moderate, low, or very low certainty, based on factors such as risk of bias, consistency, and precision. “Overbite closure” = improvement in anterior open bite/overbite; “Habit cessation” = stopping the NNS habit; “post-treatment stability” = maintaining correction without relapse; “Adverse effects” = any harms or patient-reported issues. This diagram indicates moderate certainty for bite closure outcomes but low or very low certainty for habit cessation, stability, and adverse effects, suggesting that further high-quality studies could help increase our confidence in these results.

Based on the GRADE criteria, the overall certainty of the evidence ranges from moderate for the primary outcome to low or very low for the secondary outcomes. In particular, the evidence that fixed appliances improve short-term overbite closure more than removable appliances has moderate certainty. Several studies demonstrate a consistent benefit, although this was downgraded for some risk of bias due to lack of blinding and the use of non-randomized designs in the body of evidence. In contrast, the evidence for habit cessation success and for long-term post-treatment stability has low certainty. Many of these outcomes were reported in observational studies or small trials with potential sources of bias, and definitions of “cessation” or “relapse” varied between studies. Finally, the evidence regarding the adverse effects of treatment has very low certainty, as most studies did not systematically record adverse outcomes, and the few that did were mostly descriptive. A concise summary of the GRADE ratings for each key outcome is presented in Figure 3.

Discussion

Clinical Interpretation of Main Findings

The management of NNSHs continues to be an important facet of interceptive orthodontics, given the association of persistent NNSH with malocclusions such as anterior open bite, increased overjet, and posterior crossbite. This systematic review synthesized evidence comparing fixed and removable HBAs in children with persistent digit or pacifier sucking habits. The overall findings indicate that both fixed and removable appliances can be effective in promoting habit cessation and correcting associated dentoalveolar changes; however, fixed appliances appear to achieve more rapid and consistent results, largely because they do not depend on patient compliance. Recent evidence further supports this finding: a randomized pilot study compared an extraoral electronic habit-reminder device to a fixed palatal crib, and it found higher cessation in the fixed crib arm (54.5% vs. 27.3%). However, the difference was not statistically significant due to the small sample size [29].

Mechanisms and Appliance-Specific Effects

HBAs operate primarily by interrupting the mechanical and psychological gratification associated with sucking, either through a physical barrier or a reminder effect. Fixed appliances such as the palatal crib, Bluegrass roller, and bonded spurs are cemented intraorally and function continuously, preventing the thumb or finger from creating the seal required for suction. Conversely, removable devices such as acrylic plates or removable cribs rely on the child to insert and wear them for adequate periods each day. This fundamental behavioral distinction explains much of the difference in treatment outcomes observed among studies. A Cochrane review found that children treated with orthodontic appliances were significantly more likely to stop their sucking habits than untreated controls, with fixed cribs outperforming removable arches [11]. In one comparative trial, children fitted with a fixed palatal crib were significantly more likely to stop their sucking habit than those with a passive appliance, highlighting the clear behavioral impact of fixed barriers [39]. These findings collectively suggest that fixed appliances provide a reliable mechanical deterrent and produce quicker behavioral modification.

Removable appliances, while theoretically capable of producing equivalent occlusal corrections, depend on patient motivation and parental supervision for efficacy. Several controlled trials have shown comparable dentoalveolar improvements when compliance is maintained. A Brazilian study reported that both removable and fixed palatal cribs combined with chincup therapy led to significant increases in overbite among children with anterior open bite, although fixed cribs achieved closure more rapidly [38]. A cephalometric study that assessed the changes resulting from different treatment approaches on 77 children confirmed similar results, finding that both the removable crib with chincup and bonded spur with chincup groups demonstrated greater overbite correction than controls, without significant skeletal side effects [31]. In a randomized trial using a removable palatal crib combined with a high-pull chincup, overbite improved by almost 4 mm over 12 months, and 87% of children achieved open-bite closure; the achieved correction was mainly dentoalveolar, reinforcing that removable protocols work well when compliance is adequate [37]. The comparable mechanical effect, namely, inhibition of tongue thrusting and reduction of anterior dental proclination, implies that consistency of appliance wear critically affects outcomes. However, given the notoriously variable compliance in pediatric populations, fixed devices tend to yield higher rates of success in routine clinical settings. In addition to overbite correction, fixed and removable palatal cribs may produce different arch-dimensional responses. Slaviero et al. [36] reported distinct patterns of transverse and arch-length changes between fixed and removable cribs, suggesting appliance choice can also be guided by arch-development goals in addition to compliance [36].

Patterns of Dentoalveolar and Skeletal Change

The dental and skeletal changes observed following cessation of NNSH are consistent across appliance types. Eliminating the sucking force typically results in spontaneous retroclination of the maxillary incisors, extrusion of anterior teeth, and improved vertical overlap. In studies of palatal cribs, overbite increases of 2-4 mm are common after 6-12 months of therapy [27,30]. Fixed bonded or conventional lingual spurs also lead to clinically meaningful occlusal gains. Comparative studies report overbite increases of roughly 3-4 mm over one year, with about three-quarters of children achieving positive overbite [24]. When bonded spurs were combined with high-pull chincup, the mean overbite gain exceeded 5 mm, and nearly 87% of children corrected to a positive overbite within 12 months [25]. An ultrasonographic pilot study was done in 10 children with digit-sucking habits, of whom six successfully stopped the habit using a fixed anti-digit appliance. The findings reported notable increases in overbite and trends toward normalization of masseter and orbicularis oris muscle function, although changes in fractional shortening did not reach statistical significance [35]. Another study reported that most treated patients maintained a positive overbite after crib removal, indicating stable vertical correction [28]. In removable appliance protocols, similar improvements occur when compliance is optimal, but relapse is more frequent if the habit is only partially suppressed. The present analysis supports the notion that cessation rather than appliance type per se determines the magnitude of occlusal improvement.

The quantitative meta-analysis demonstrates that both Giuntini et al. (2008) and Slaviero et al. (2017) found removable appliances to produce greater mean overbite changes than fixed appliances [30,36]. However, neither study individually achieved statistical significance, as evidenced by both CIs crossing zero. When these studies are combined through meta-analysis using the inverse variance random-effects method, the pooled effect was -0.75 mm (95% CI = -1.57 to 0.07), weighted heavily toward the more precise Giuntini et al. study (60% contribution). This pooled estimate maintained the same direction (favoring removable) but with reduced uncertainty compared to each study alone, yet it still crossed zero and failed to achieve statistical significance at the p < 0.05 level (p = 0.07). The heterogeneity assessment revealed zero between-study variation (I² = 0%), indicating perfect concordance in the direction and approximate magnitude of effects despite the different methodological approaches of the studies.

The numerical outcomes shown in Table 3 and Figure 2 corroborate the qualitative findings, highlighting the modest but clinically meaningful advantage of fixed appliances for vertical bite closure. Although individual studies varied in appliance design and follow-up duration, the consistency of positive mean differences supports the mechanical superiority of fixed deterrent designs. These data also align with the meta-analysis conclusions by Meng et al. [40], who reported comparable pooled gains of approximately 2 mm in overbite, favoring fixed cribs and bonded spurs [40]. Within fixed protocols, palatal cribs may deliver more consistent closure than spurs; one randomized study found that fixed cribs led to 100% positive overbite after 12 months versus ~54% with bonded spurs, even though both improved open bite clinically [32].

Stability and Relapse

Treatment stability is another major consideration. As fixed appliances are clinician-controlled, they ensure continuous deterrence until the habit has been extinguished for a sustained period, reducing the risk of early discontinuation. Longitudinal data evaluating the long-term stability of quad-helix/crib treatment in subjects with dentoskeletal open bite demonstrated stable overbite correction five years after treatment [33]. Cassis et al. [18] also observed that 96% of patients treated with bonded spurs and a chincup retained positive overbite three years post-treatment, underscoring the durability of outcomes when fixed devices are used until complete behavioral extinction [18]. Importantly, the cohort had a low relapse rate (4%), which was not influenced by initial severity or amount of correction; this suggests that once the habit is eliminated early, stability is generally maintained through growth [26]. In contrast, removable appliances present a higher risk of relapse because they may be discontinued prematurely or inconsistently.

Other important clinical considerations that influence appliance selection are patient comfort, oral hygiene, and acceptance. Although fixed devices remove the issue of compliance, they can provoke transient discomfort, speech disturbance, and mucosal irritation. However, most children adapt quickly to fixed spurs, with the majority reporting discomfort for less than one week; bonded spurs, in particular, showed better chewing and eating acceptance than conventional banded designs [24]. Removable appliances, by contrast, are generally more comfortable and facilitate easier cleaning, but they may impede speech during wear and are prone to being misplaced or broken. While both fixed and removable approaches have high levels of child and parent acceptance in trials, each has unique issues with maintenance: fixed cribs may require re-cementation due to dislodgement, whereas removable reminder devices or plates may break or be inconsistently worn [29]. In addition, the psychosocial aspect should not be underestimated: for self-conscious or anxious children, a less conspicuous removable device may be more acceptable, whereas uncooperative or younger children may benefit from a fixed appliance that functions passively without daily intervention. Hence, the ideal approach remains patient-specific and requires clinicians to balance compliance capacity with comfort and aesthetic considerations.

Adjunctive Behavioral and Myofunctional Strategies

Behavioral reinforcement remains a critical adjunct to any mechanical intervention. In NNSH, behavioral modification complements the mechanical deterrent effect of appliances and addresses the emotional and habitual components of sucking behaviors. Studies consistently demonstrate that combining HBAs with psychological or parental reinforcement strategies enhances cessation rates. The efficacy of positive reinforcement in habit reversal has been documented in historical texts [41], and contemporary reports affirm that parental involvement, encouragement, and reward systems expedite cessation and reduce relapse. Dentists should therefore integrate motivational counseling into treatment plans, set achievable targets, and involve caregivers in monitoring progress.

Recent evidence also points to adjunctive myofunctional therapy as a valuable component in achieving functional normalization once the habit ceases. A study demonstrated that orofacial myofunctional therapy improved tongue posture and occlusal contact following cessation of digit sucking, supporting its role in stabilizing outcomes once the habit is broken [42]. Functional rehabilitation may prevent residual tongue thrusting or abnormal swallowing patterns that could otherwise perpetuate open-bite relapse. Similarly, another study reported that myofunctional appliances increased lip strength and improved swallowing coordination, although these alone were insufficient to treat an open bite without habit elimination. Functional rehabilitation via orofacial myofunctional therapy or appliances such as the “Froggy Mouth” may prevent residual tongue thrusting that could otherwise jeopardize the orthodontic correction [43]. Thus, combining mechanical habit interruption with functional re-education provides a comprehensive framework for long-term stability.

Economic and accessibility factors, though infrequently discussed, also influence appliance selection. Fixed appliances may require laboratory fabrication and longer chair time, potentially increasing costs; however, they are rarely lost or replaced. Hence, cost considerations should not deter clinicians from employing fixed devices when compliance is a concern, as the potential for early cessation and reduced relapse can offset the initial expense.

Limitations, evidence gaps, and future research

Despite the strong consensus that fixed appliances deliver superior predictability, the quality of evidence across the literature remains moderate to low. Many studies are small-scale, single-center trials with heterogeneous designs, varying follow-up durations, and differing outcome measures. A meta-analysis concluded that bonded spurs, fixed, and removable cribs all significantly improve anterior open bite, with no statistically significant difference in overall dentoskeletal outcome [40]. This apparent equivalence, however, should be interpreted cautiously: while morphological outcomes may be similar, fixed devices still demonstrate higher rates of confirmed habit cessation, a behavioral endpoint not always captured in orthodontic metrics. The lack of standardized measures of habit cessation and relapse across studies limits definitive comparison. Similar methodological constraints also contribute to downgrades in certainty, including projected control data in some trials and pilot-level underpowering in others [29,32]. A study found that adding orofacial myofunctional therapy significantly reduced open-bite relapse after orthodontic correction, underscoring the importance of addressing oral habits in retention [44]. Future investigations should therefore employ uniform behavioral and occlusal outcome definitions and extend follow-up beyond active treatment to assess true long-term stability. The heterogeneity in patient age, habit intensity, and coexisting functional disturbances further complicates interpretation. Younger patients often exhibit faster adaptation and more robust spontaneous correction once the habit stops, as alveolar growth potential remains high [45]. In older children, dentoalveolar compensation may be less pronounced, and adjunctive orthodontic or myofunctional therapy is necessary. Accordingly, appliance selection should account for developmental stage and skeletal maturity, with early intervention preferred whenever possible.

Another limitation of the current evidence is the under-reporting of adverse effects and patient-centered outcomes. Few studies systematically assess discomfort, speech interference, or quality of life outcomes. Where recorded, most side effects were mild and transient. Multiple trials on both fixed and removable appliances did not report any serious adverse events. The unwanted effects were mainly transient speech difficulties, gingival irritation, and appliance breakage or dislodgement requiring repair [24,29,34,37]. Furthermore, almost no studies evaluated psychosocial aspects of prolonged appliance wear, yet these factors critically influence adherence and perceived success. Given the modern emphasis on patient-reported outcomes in pediatric dentistry, future research should incorporate validated comfort and satisfaction questionnaires to inform balanced clinical decisions.

Clinical implications

From a clinical perspective, the cumulative evidence supports a pragmatic approach to NNSH management. Fixed HBAs are recommended when rapid, reliable cessation is required or when compliance is uncertain. They ensure constant deterrence and predictable correction of open bite and incisor inclination. Removable appliances remain appropriate for older, cooperative children or when aesthetic or speech considerations favor an intermittent approach. In both modalities, clinician monitoring and behavioral reinforcement are crucial. Appliances should remain in place for several months after the last reported habit episode to minimize relapse, and retention or functional follow-up should continue until the eruption of permanent incisors has stabilized the occlusion.

## Conclusions

Both fixed and removable orthodontic HBAs can play a valuable role in stopping harmful sucking habits and correcting their dental sequelae. The current literature indicates that, when worn as prescribed, removable appliances can achieve overbite correction and habit cessation rates comparable to those of fixed cribs. Fixed appliances, however, tend to produce slightly more skeletal and tooth-extrusive effects in many studies. They also obviate the problem of non-compliance, at the expense of potential discomfort and hygiene issues. Thus, neither modality is universally superior; rather, the choice should be guided by patient cooperation and the specific orthodontic goals. In practical pediatric settings, fixed appliances are often preferred for younger or less compliant patients, while removable devices may suffice for older or highly motivated children. In all cases, appliances should be combined with behavioral guidance for the best results. Ultimately, the evidence underscores that careful selection of an HBA, whether fixed or removable, can significantly improve outcomes in children with persistent non-nutritive sucking, and it should be part of the clinician’s armamentarium for early habit management.
